# PD193 Submission Processes And Requirements For Health Technology Assessment In Australia, Canada, England, France, And Germany

**DOI:** 10.1017/S026646232400415X

**Published:** 2025-01-07

**Authors:** Emily Gregg, Charlotte Graham, Karina Watts, Stuart Mealing

## Abstract

**Introduction:**

As health technology assessment (HTA) bodies introduce more rigorous requirements, the submission process is becoming increasingly diverse between countries. This study assessed the HTA submission processes and requirements in Australia, Canada, England, France, and Germany. This helps identify where efficiencies can be made in the global market access strategy, such as when to submit HTA dossiers.

**Methods:**

A pragmatic review and desk-based research were conducted in November 2023. Published articles, HTA guidelines, process documents, conference abstracts, and white papers were reviewed to identify country-specific processes with implications for market access strategy. Where available, information was extracted about the general submission process and stakeholders involved (including regulatory, HTA, and pricing authorities), clinical evidence requirements, and pharmacoeconomic evidence requirements for HTA submission. Comparisons of the median time from marketing authorization to HTA decision within each country allowed the identification of efficiencies in individual HTA submission processes. The key findings and between-country differences were summarized narratively.

**Results:**

The review identified several areas with implications for market access strategy. The median HTA review time was shortest in Australia (125 days) and longest in England (266 days). Australia and Canada have both sequential and parallel regulatory and HTA processes. The median time taken from regulatory approval to HTA recommendation was faster with the parallel process than with the sequential process in both countries. All countries required comparative clinical evidence within the indication. The weight placed on pharmacoeconomic evidence varied between countries. In Germany, economic evaluation has yet to play a real role. Requirements for additional information after HTA submission occurred within all HTA bodies.

**Conclusions:**

HTA processes in Australia, Canada, England, France, and Germany differ from one another. This will likely affect the market access strategy for health technology developers. Similar requirements allow efficiencies in the preparation of submission documentation. Future research should investigate the impact of the European Union HTA regulation on market access and how this could affect strategic decision-making.

